# Research on UAV Path Planning Based on Enhanced Artificial Lemming Algorithm

**DOI:** 10.3390/biomimetics11050297

**Published:** 2026-04-24

**Authors:** Yu Liu, Maosheng Fu, Chaochuan Jia, Zhengyu Liu, Xuemei Zhu, Bao Zhou, Jingya Zhang, Hai Liu

**Affiliations:** 1 School of Electronic Information and Artificial Intelligence, West Anhui University, Lu’an 237012, China; 2 Anhui Province Intelligent Hydraulic Machinery Joint Construction Subject Key Laboratory, Lu’an 237012, China; 3 Experimental Training Teaching Management Department, West Anhui University, Lu’an 237012, China; 4 School of Physics and Electronic Engineering, Hubei University of Arts and Science, Xiangyang 441053, China

**Keywords:** UAV, path planning, artificial lemming algorithm, BetaCOBL

## Abstract

Unmanned aerial vehicle path planning faces multiple challenges in terms of effectiveness and safety. Traditional optimization methods are difficult to use to effectively find the best route. An enhanced artificial lemming optimization algorithm (ALAEN) is proposed here, which introduces stochastic differential mutation and Beta opposition-based learning into the artificial lemming algorithm (ALA). The comparison with other algorithms on the CEC2017 test set shows that it can effectively improve the optimization ability and convergence speed of the artificial lemming algorithm. Among all algorithms, ALA has an overall ranking of 5.45 and ALAEN has a ranking of 1.34. The ability of ALAEN to solve the actual problem of UAV trajectory planning is tested on two different maps, and it is found that it can effectively improve the path planning ability and ensure safety compared with the ALA. In the small map scene, the average cost function of ALA is 92.999, and the average cost function of ALAEN is 91.598, which is a significant improvement. Compared with other algorithms, ALAEN has the shortest trajectory route and trajectory cost function.

## 1. Introduction

With the development of unmanned aerial vehicle (UAV) technology, its application in military reconnaissance, logistics distribution, agricultural plant protection, emergency rescue and other fields is becoming more and more extensive [1,2,3,4]. However, the efficient and safe flight of UAVs in complex environments faces many challenges, among which the path planning problem is particularly critical. The goal of path planning is to plan an optimal path from the starting point to the end point for the UAV while meeting the mission requirements and flight constraints while avoiding collisions with obstacles. This problem involves multiple variables, constraints and objective functions. Traditional optimization methods often find it difficult to find a satisfactory solution within a reasonable time [5,6,7].

Recently, the research on UAV path planning has been mainly based on optimization algorithms. There are two main types of algorithms: classical and heuristic optimization algorithms. Classic optimization algorithms include the A* algorithm, Dijkstra algorithm, rapid exploration random tree (RRT) algorithm, etc. [8,9,10]. The research based on these algorithms includes the improved A* algorithm with a two-way mechanism, the RRT algorithm based on a rolling window, the joint A* algorithm and RRT algorithm and so on [11,12,13]. Although these algorithms perform well in simple environments, they often face problems such as low computational efficiency and are prone to falling into local optimality in complex three-dimensional environments.

Heuristic optimization algorithms have gradually become the mainstream method for solving UAV path planning problems due to their strong global search capabilities, high adaptability, and low computational complexity [14,15,16]. These algorithms can find the global optimal solution in a complex search space by simulating biological behaviors or physical processes in nature. At present, the research on optimizing UAV trajectory based on heuristic optimization algorithms mainly focuses on improving the optimization algorithm with joint strategies, such as the hybrid enhanced particle swarm optimization algorithm [17], the enhanced dung beetle optimization algorithm with joint strategy [18,19], the improved chaos sparrow search algorithm (SSA) [20], the differential evolution algorithm based on multi-strategy improvement [21], the improved marine predator algorithm [22], etc. Other studies are centered on fusion algorithms, such as the gray wolf algorithm (GWO) integrated with the artificial potential field method [23] and the hybrid algorithm based on the GWO and differential evolution (DE) [24]. Studies have shown that improved or fused heuristic optimization algorithms can effectively find the global optimal solution and can effectively solve the UAV path planning problem. Nevertheless, existing optimization algorithms still face some challenges in UAV path planning. For example, how to improve the convergence speed of the algorithm and the accuracy of the solution while ensuring the smoothness of the path; how to adjust the path planning strategy in real time in a dynamic environment to cope with environmental changes; and how to achieve efficient path planning in multi-UAV collaborative tasks to improve mission efficiency and safety. Therefore, researching and developing more efficient and intelligent optimization algorithms is of great theoretical and practical significance in the UAV path planning application.

The artificial lemming algorithm (ALA) simulates four behaviors of lemmings (long-distance migration, burrowing, foraging, and escaping from natural enemies), which correspond to different exploration and utilization strategies [25]. This diverse strategy makes ALA more adaptable to complex optimization problems. To increase the chance of finding the global optimal solution and improve the accuracy of the algorithm, ALA introduces Brownian motion and Levy flight. This combination makes ALA perform well in dealing with high-dimensional and non-convex optimization problems. Some research has also been conducted recently regarding the variants of ALA. Tang et al. introduced a second-order Bernstein polynomial and chaotic mapping function initialization strategy into ALA and achieved a certain performance boost [26]. Zhu et al. significantly resolved the problem of becoming trapped in local optima by introducing three strategies into ALA (EALA), including differential mutation [27]. However, its global optimization ability and convergence speed are still insufficient.

To solve the problems of slow convergence speed and poor ability to find the optimal solution of the ALA, an enhanced artificial lemming algorithm (ALAEN) was proposed. The ALAEN can greatly enhance the optimization ability and convergence speed of ALA by introducing stochastic differential mutation (SDM) and Beta opposition-based learning (BetaCOBL) and can effectively avoid falling into local optimal solutions [28,29]. Through the analysis of the test results of the CEC2017 test function by multiple algorithms, it is found that ALAEN enhanced by the joint strategy can improve the algorithm’s optimization ability and convergence speed effectively. In the UAV trajectory planning experiment, it is found that the ALAEN can find the shortest path and the lowest cost function.

This paper is organized as follows: Section 2 is the theoretical basis, which elaborates on the mathematical model of 3D UAV trajectory planning and the optimization process of the ALAEN. Section 3 is the experimental results analysis part, which first discusses the CEC2017 function set test results in detail and then constructs two map models to test the algorithm’s ability in UAV trajectory optimization. The last section provides a summary and outlook.

## 2. UAV Flight Optimization Problem

This section aims to elaborate on the process of the UAV trajectory optimization problem. First, the mathematical model of UAV flight optimization will be explained, including environmental map modeling and trajectory planning. Secondly, the inspiration background and mathematical model of the ALAEN will be introduced. Finally, the process of ALAEN optimizing UAV trajectory will be described in detail.

### 2.1. UAV Path Planning

#### 2.1.1. Environment Modeling

To establish an environmental model is the most important thing to do in the UAV path planning problem, which is the basis and premise for testing whether the UAV can complete a specific terrain task. The baseline terrain characteristics will be simulated using Equation (1) [30].

(1) $$\begin{array}{l} z(x,y)=\sin(y+a_{1})+a_{2}\sin(x)+a_{3}\cos\left(a_{4}\sqrt{y^{2}+x^{2}}\right)\\ +a_{5}\cos(y)+a_{6}\sin\left(a_{6}\sqrt{y^{2}+x^{2}}\right)+a_{7}\cos(y) \end{array}$$

Among them, *x*, *y*, and *z* are the coordinates of a point on the terrain. *a*_1_ to *a*_7_ are constant coefficients, and their sizes can be changed to obtain different landform features. Overlaying the mountain peak model on the baseline terrain can obtain the obstacle avoidance area. The peak is obtained by Equation (2).

(2) $$h(x,y)=\sum_{i}h_{n}\exp\left[-\frac{{\left(x-x_{n}\right)}^{2}}{a_{n}^{2}}-\frac{{\left(y-y_{n}\right)}^{2}}{b_{n}^{2}}\right]+h_{b}$$

*h*_b_ and *h*_n_ represent the height of the base terrain and the *n*th peak respectively; (*x*_n_, *y*_n_) represents the center coordinate position of the *n*th peak; and *a*_n_ and *b*_n_ represent the slope of the nth peak along the *x*-axis and *y*-axis respectively. From Equations (1) and (2), the environmental terrain formula can be obtained as shown in Equation (3).

(3) Z(x,y)=max[z(x,y),h(x,y)]

**Figure 1:**
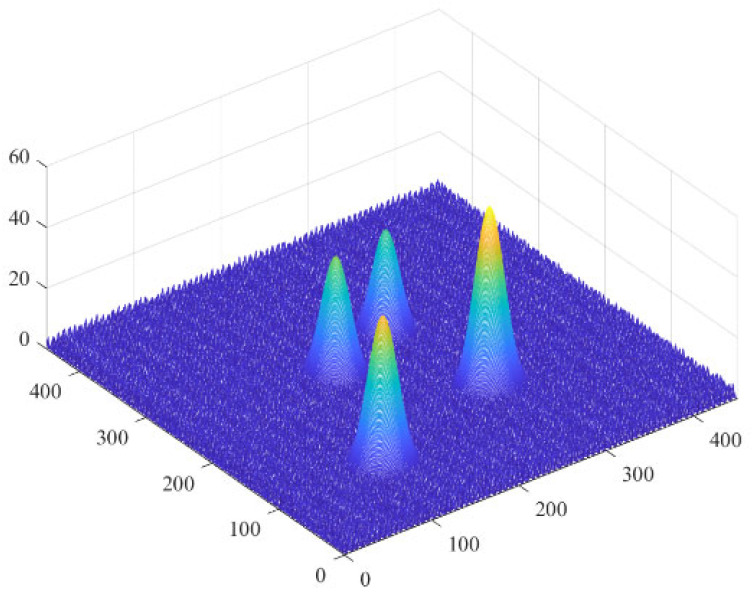
Obstacle-free terrain map.

The obstacle-free terrain map is shown in Figure 1.

**Figure 2:**
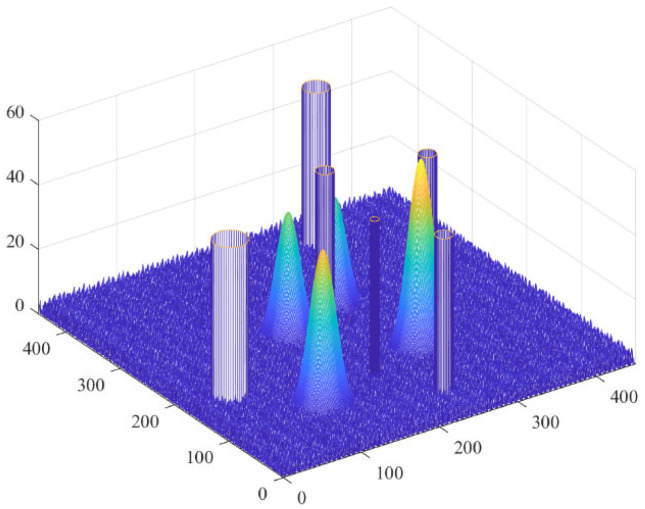
Terrain map with obstacles.

While avoiding obstacles, drones often encounter threat areas that affect the flight safety of the drones. These threat areas can be detection threat areas of enemy radars and air defense missile systems. Once a drone enters these areas, it is likely to be shot down or crash. Here, a cylindrical area is used to represent the threat area, and the threat capability decreases from the center position outward. In summary, the final simulation effect is shown in Figure 2, where the cylinder is the threat area.

#### 2.1.2. UAV Trajectory Planning Modeling

A reasonable track evaluation function can ensure that the final result meets the requirements and ensures that the track is valid. In the actual environment, drones need to constantly adapt to the changing environment, so in the process of drone path planning, the optimal path will appear to be more complex and contain many different features. In this paper, the main factors affecting drone performance include track length, flight altitude, maximum climb angle, etc.

In practical applications, the shortest path is closely related to the best path. In the process of UAV trajectory planning, the trajectory length is the primary factor to be considered in trajectory planning. A shorter route means lower energy consumption and shorter time, and the probability of being discovered by unknown threats is also lower. Assume that a route has *i* nodes, where the distance between the *n*th waypoint and the (*n* + 1)th waypoint is expressed as *l*_n_, and the coordinates of these two waypoints are expressed as (*x*_n_, *y*_n_, z_n_) and (*x*_n+1_, *y*_n+1_, z_n+1_) recorded as *g*(*n*) and *g*(*n* + 1). The path needs to satisfy Equation (4) [31].

(4) $$\left\{\begin{array}{l} l_{n}=∥g(n+1)-g(n){∥}_{2}\\ L_{path}=\sum_{n=1}^{i-1}l_{n} \end{array}\right.$$

During the flight, if the drone cannot avoid obstacles or flies into a threat area, it will face the risk of being shot down or crashing and thus cannot reach the destination, which is recorded as $L_{path}=\infty$. However, infinite functions are difficult to represent in practical problems, so a penalty method will be used to deal with them.

The flight altitude setting of a drone needs to avoid threats from unknown radars and collisions with mountains and the ground, so the stable flight altitude should not be too high or too low. And the flight altitude should not fluctuate too much. A stable flight altitude can reduce the burden on the control system and save more fuel. In order to make drone flight safer, the flight altitude model given by Equation (5) is used.

(5) $$\left\{\begin{array}{l} h_{height}=\sqrt{\frac{1}{i}\sum_{n=0}^{i-1}(z(n)-\bar{z}{)}^{2}}\\ \bar{z}=\frac{1}{i}\sum_{n=0}^{i-1}z(i) \end{array}\right.$$

Among them, $h_{height}$ is the standard deviation cost function of height, and $\bar{z}$ is the average value of height.

Another factor that affects the operability of the drone is the angle cost function limit. During the flight, the angle of the drone needs to be less than the preset maximum angle, and the size of the angle will affect its flight stability. Set the maximum angle to ϕ, the current angle to *θ* and *a*_n_ is the nth route segment vector. The mathematical model is shown in Equation (6) [32].

(6) $$\left\{\begin{array}{l} \cos\theta =\frac{a_{n}^{T}a_{n+1}}{\left|a_{n}||a_{n+1}\right|}\\ J_{turn}=\sum_{i=1}^{n}\left(\cos\phi -\cos\theta_{n}\right) \end{array}\right.$$

Through Formulas (4)–(6), the cost function of UAV trajectory planning can be obtained, and the trajectory evaluation function is shown in Equation (7).

(7) $$J_{cost}=w_{1}L_{path}+w_{2}h_{height}+w_{3}J_{turn}$$

Among them, *J*_cost_ is the total cost function, the parameters *w*_1_, *w*_2_ and *w*_3_ represent the weight of each cost function, and satisfy the conditions as shown in Formula (8).

(8) $$\left\{\begin{array}{l} w_{n}\geq 0\\ \sum_{i=1}^{3}w_{n}=1 \end{array}\right.$$

By effectively processing the total cost function, a track composed of line segments can be obtained. It is undeniable that the obtained path is often only feasible in theory, but in order to be actually flyable, it is necessary to smooth the track. This paper uses the interpolation method to call the B-spline curve function to smooth the obtained track to achieve the purpose of actual flyability.

### 2.2. ALAEN

#### 2.2.1. Initialization

The ALAEN, like other population-based algorithms, randomly initializes the positions of all agents. The set of all initial candidate solutions is a matrix $\vec{A}$ consisting of *N* (population size) rows and *D* (number of dimensions) columns as shown in Formula (9). The current optimal solution is the best position in each iteration. The decision variable $a_{i,j}$ for each dimension is calculated by Equation (10).

(9) $$\vec{A}=\left[\begin{array}{ccccc} a_{1,1} & a_{1,2} & \cdots & a_{1,D-1} & a_{1,D}\\ a_{2,1} & z_{2,1} & \cdots & a_{2,D-1} & a_{2,D}\\ \cdots & \cdots & a_{i,j} & \cdots & \cdots \\ \vdots & \vdots & \vdots & \vdots & \vdots \\ a_{N-1,1} & a_{N-1,2} & \cdots & a_{N-1,D-1} & a_{N-1,D}\\ a_{N,1} & a_{N,2} & \cdots & a_{N,D-1} & a_{N,D} \end{array}\right]$$

(10) $$a_{i,j}=L_{j}+r\times (U_{j}-L_{j}),\;i=1,2,\cdots ,N,\;j=1,2,\cdots ,D$$

where *r* is a random value in the range (0, 1), $L_{j}$ and $U_{j}$ represent the lower and upper limits of the *j*-th dimension, respectively.

#### 2.2.2. Exploration

During the exploration phase, lemmings have two behaviors: long-distance migration due to food shortages and digging to provide themselves with safe shelters and food storage space. During migration, lemmings will explore the search space based on their own location and the other individuals, with the goal of finding a habitat with sufficient food resources. In addition, due to the combined effects of multiple factors such as the ecological environment and food distribution, the direction and distance of lemmings’ movement during migration will be dynamically adjusted according to actual conditions. The migration behavior is modeled using Equation (11).

(11) $${\vec{A}}_{i}(t+1)={\vec{A}}_{best}(t)+S\times \vec{BM}\times (\vec{R}\times ({\vec{A}}_{best}(t)-{\vec{A}}_{i}(t))+(1-\vec{R})\times ({\vec{A}}_{i}(t)-{\vec{A}}_{a}(t)))$$

Among them, ${\vec{A}}_{i}(t+1)$ represents the position of the *i*-th search agent at the *t* + 1 iteration, and ${\vec{A}}_{best}(t)$ represents the current optimal solution. *S* is the signal to change the search direction, which is obtained by Equation (12). $\vec{BM}$ represents a random number vector that represents Brownian motion, as shown in Equation (13). *R* is a vector of size 1 × D, which is generated by Formula (14). ${\vec{A}}_{i}(t)$ stands for the current position of the *i*-th search agent. ${\vec{A}}_{a}(t)$ stands for a random search individual, and a is between 1 and N.

(12) $$F=\left\{\begin{array}{ll} 1 & if\left\lfloor 2\times r+1\right\rfloor =1\\ -1 & if\left\lfloor 2\times r+1\right\rfloor =2 \end{array}\right.$$

(13) $$f_{BM}(x;0,1)=\frac{1}{\sqrt{2\pi }}\times exp\left(-\frac{x^{2}}{2}\right)$$

(14) $$\vec{R}=2\times r(1,D)-1$$

$\left\lfloor \cdot \right\rfloor$ represents the floor function.

In the digging behavior, lemmings will dig new burrows based on the location of the current burrow and the location of other individuals. This behavior pattern helps them quickly avoid the threat of predators and find food more efficiently. This behavior is simulated by Formula (15).

(15) $${\vec{A}}_{i}(t+1)={\vec{A}}_{i}(t)+F\times L\times ({\vec{A}}_{best}(t)-{\vec{A}}_{b}(t))$$

where *L* is a random number related to the current iteration and is calculated by Equation (16). ${\vec{A}}_{b}$ stands for a random search individual, and *b* is a random integer in (1, *N*). *L* and ${\vec{A}}_{b}$ are used to describe the interaction between individuals when digging new burrows.

(16) $$L=r\times (1+sin(\frac{t}{2}))$$

#### 2.2.3. Exploitation

During the exploitation phase, lemmings also have two behaviors. The first is random foraging behavior. Lemmings establish a foraging area within their habitat based on the amount and availability of food and wander randomly within this area. When modeling this phase, the spiral winding mechanism is considered, as shown in Formula (17).

(17) $${\vec{A}}_{i}(t+1)={\vec{A}}_{best}(t)+F\times spiral\times r\times {\vec{A}}_{i}(t)$$

Here, spiral represents the search shape during foraging, which is obtained by Equations (18) and (19).

(18) spiral=radius×(sin(2π×r)+cos(2π×r))

(19) $$radius=\sqrt{\sum_{j=1}^{Dim}{\left(z_{best,j}(t)-z_{i,j}(t)\right)}^{2}}$$

The second type is predator avoidance behavior, which focuses on the behavior of lemmings when they face danger. Once they detect an enemy, lemmings will use their excellent running ability to escape back to their caves. Simultaneously, lemmings will also make confusing movements to avoid being hunted by predators. The corresponding mathematical expression is shown in Formula (20).

(20) $${\vec{A}}_{i}(t+1)={\vec{A}}_{best}(t)+F\times R\times Levy(D)\times ({\vec{A}}_{best}(t)-{\vec{A}}_{i}(t))$$

(21) $$G=2\times (1-\frac{t}{T_{max}})$$

where *R* is the escape coefficient, denotes their escape ability, which decreases with the increase in the number of iterations, see Equation (21). *T* represents the maximum number of iterations. And Equation (22) is the Levy flight function, which is used to simulate the confusing movements of lemmings during their escape. The expression of the Levy flight function is as follows:

(22) $$Levy(x)=0.01\times \frac{u\times \sigma }{|\nu {|}^{\frac{1}{\beta }}},\sigma ={\left(\frac{\Gamma (1+\beta )\times sin(\frac{\pi \beta }{2})}{\Gamma (\frac{1+\beta }{2})\times \beta \times 2^{\left(\frac{\beta -1}{2}\right)}}\right)}^{\frac{1}{\beta }}$$

#### 2.2.4. Stochastic Differential Mutation

The stochastic differential mutation (SDM) strategy uses the current individual, the current optimal individual, and a random individual selected from the population to perform stochastic differences to obtain a new individual. The specific expression is shown as Equation (23).

(23) $$A(t+1)=rand\times (A_{best}(t)-A(t))+rand\times (A^{\prime }(t)-A(t))$$

where *rand* is a random number in [0, 1], $A^{\prime }$ is the position of the random individual. In each iteration, the SDM strategy (i.e., Equation (23)) is used to perturb the population and generate new individuals.

#### 2.2.5. BetaCOBL

BetaCOBL uses the Beta distribution to calculate a pair of opposite solutions, namely concave and convex opposite solutions. Concave and convex opposite solutions refer to solutions generated based on a probability density function in which the opposite point in the concave opposite solution has the highest probability of being selected, and conversely, the corresponding point in the convex opposite solution has the lowest probability of being selected. BetaCOBL estimates the population diversity before performing concave and convex adversarial learning. If the population diversity is above a predefined threshold, BetaCOBL adopts a (*μ* + *λ*) selection strategy for all primal solutions in the population; otherwise, it adopts a (*μ*, *λ*) selection strategy for the worst half of the primal solutions in the population. Therefore, BetaCOBL can apply one of the two selection operators based on the convergence progress, thus avoiding the waste of fitness evaluation.

The concave inverse solution is calculated by a Beta distribution with both *α* and *β* larger than 1, as shown in Equations (24)–(29).

(24) $${\tilde{a}}_{i,j}=\left(L_{j}-U_{j}\right)\cdot Beta(\alpha ,\beta )+a_{i,j}$$

(25) $$\alpha =\left\{\begin{array}{ll} s\cdot peak & ifmode<0.5\\ s & otherwise \end{array}\right.$$

(26) $$\beta =\left\{\begin{array}{ll} s & ifmode<0.5\\ s\cdot peak & otherwise \end{array}\;\right.$$

(27) $$s={\left(\frac{1}{\sqrt{normDiv}}\right)}^{1+N(0,0.5)}$$

(28) $$peak=\left\{\begin{array}{ll} \frac{(s-2)\cdot model+1}{s\cdot (1-mode)} & ifmode<0.5\\ \frac{2-s}{s}+\frac{s-1}{s\cdot mode} & otherwise \end{array}\right.$$

(29) $$mode=\frac{\left(U_{j}+L_{j}-a_{i,j}(t)\right)-U_{j}}{L_{j}-U_{j}}$$

where Beta (α, β) and N (0, 0.5) represent a Beta distribution with parameters α and β and a Gaussian distribution with mean 0 and variance 0.5, respectively. In addition, the normalized diversity represented by normDiv is calculated as follows:

(30) $$normDiv=\frac{1}{NP}\sum_{i=1}^{NP}CD\left(a_{i}(t),P(t)\right)$$

(31) $$CD\left(a_{i}(t),P(t)\right)=min_{c\in P(t),c\neq a_{i}(t)}d\left(c,a_{i}(t)\right)$$

(32) $$d\left(c,a_{i}(t)\right)=\sqrt{\frac{1}{D}\sum_{j=1}^{D}{\left(\frac{a_{i,j}(t)-c_{j}}{L_{j}-U_{j}}\right)}^{2}}$$

The formula for calculating the convex inverse solution is the same as the formula for calculating the concave inverse solution, except that the *mode* and *s* are calculated by Equations (33) and (34).

(33) $$mode=\frac{a_{i,j}(t)-U_{j}}{L_{j}-U_{j}}$$

(34) $$spread=0.1\cdot \sqrt{normDiv}+0.9$$

#### 2.2.6. Mode Transition

In ALAEN, the choice of these four search modes is related to the energy level of the lemmings. To maintain a good balance between exploration and exploitation, an energy factor is devised to gradually decrease during the iteration process. When the individuals have enough energy, they will enter the exploration phase; otherwise, they will proceed to the exploitation phase. The energy factor is obtained by Equation (35).

(35) $$E(t)=4\times \arctan\left(1-\frac{t}{T_{max}}\right)\times \ln\left(\frac{1}{rand}\right)$$

As the iteration proceeds, *E* will fluctuate and drop to 0 progressively. When the threshold is 1, during the iteration process, the chances of lemmings exploring and developing are almost the same. And the probability of *E* being greater than 1 is 0.5. The pseudocode for ALAEN is shown in Algorithm 1.
**Algorithm 1.** The pseudo code of the ALAENInput: maximum iterations numbers *T*, population *N* and dimension *D*;
Output: global optimal solution
${\vec{A}}_{best}$ and its fitness value *f_b_*_est_; Randomly initialize the population; Evaluate the fitness values of all lemming individuals;
Record the current optimal solution ${\vec{A}}_{best}$;
*t* = 1;
**while** *t* ≤ *T* **do**
        Use formula (34) to calculate the value of *E*;
        ***for*** each search individual ${\vec{A}}_{i}$ **do**
                ***if*** E > 1 then
                        Exploration phase; **                        *if*** rand < 0.3 **then**
                                Update the current position by Equation (10);
                        **else**
                                Update the current position by Equation (14);
                        **end**
                **else**
                        Exploitation phase;
                        ***if*** rand < 0.5 **then**
                                Update the current position by Equation (16);
                        **else**

                                Update the current position by Equation (19);
                        **end**
                **end**
                Update the current position by Equation (22);
        **end**
        Recalculate the fitness values of all lemming individuals;
        Update the optimal solution ${\vec{A}}_{best}(t)$ obtained so far;
        use BetaCOBL to update the current position;
        *t* = *t* + 1;
**end**

### 2.3. Track Optimization Based on ALAEN

In this section, we will construct the corresponding solution ideas and give the algorithm optimization process based on the enhanced ALA and the total cost function of the track introduced in the previous article so as to achieve the optimization of the three-dimensional UAV track. Combined with the characteristics of the ALAEN, the specific implementation process of the three-dimensional UAV flight optimization is shown below.

1. Parameter initialization. Initialize the required parameters, such as the weight in the track evaluation function and the setting of the maximum rotation in the UAV’s own constraints.
2. Initialize the population, such as setting the number of lemming populations.
3. Set the take-off point and target point of the UAV, the location of the threat area, and record the current environmental information.
4. Use ALAEN to optimize and update the track evaluation function and determine the candidate path through waypoint search.
5. Update the path. If the current track point is better than the previous one, reconstruct a new waypoint to obtain the best path.
6. Determine whether the termination condition is met. If not, go back to the fourth step; otherwise, continue to the next step.
7. The algorithm ends when convergence or the maximum iterations is reached, and the optimal track route is obtained.

### 2.4. Ablation Experiment

**Figure 3:**
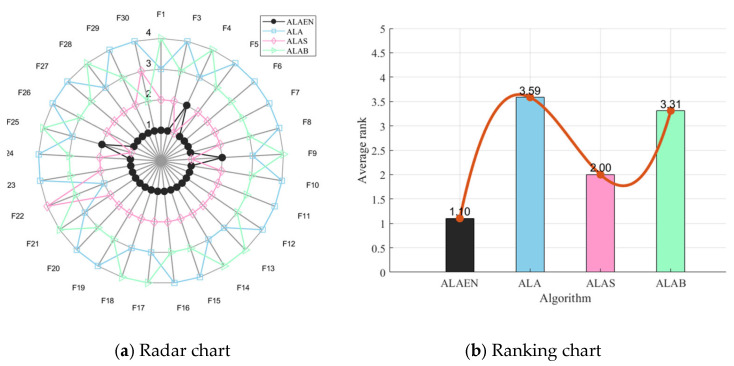
Radar and ranking charts for CEC2017 (Ablation Experiment).

Since the ALAEN incorporates two strategies based on the original algorithm, it is necessary to analyze the selection of these strategies before comparing it with other algorithms. Among them, ALAB represents the algorithm integrated with BetaCOBL, and ALAS represents the algorithm with SDM. CEC2017 basic test functions are selected as the test objects. Figure 3 shows the radar chart and sorting chart of the four algorithms. The sorting method is to rank the algorithm on each function and then take the average. As shown in Figure 3, both strategies played a crucial role. The ALAB and ALA outperformed the ALA, ranking 3.31 and 2.00 respectively. The ALAEN, which combines the two strategies, ranks 1.10. This demonstrates that the joint strategy can effectively improve the search and convergence capabilities of the algorithm.

## 3. Experiment Results and Analysis

The simulation platform runs on a Windows 11-based computer equipped with a Core (TM) i9—14,900 K, 16 GB memory, and a dedicated graphics card (RTX5080). All algorithms are implemented in MATLAB R2024a.

### 3.1. CEC2017 Test Functions

The CEC2017 standard test function will be used to test the comprehensive performance of ALAEN and other algorithms. Currently, the CEC2017 test set has 29 test functions, which introduce composite functions and hierarchical structure problems. The starfish optimization algorithm (SFOA) [33], L-SHADE algorithm [34], EALA, particle swarm optimization (PSO) [35], SSA [36], GWO [37] and Harris hawks optimization (HHO) [38] are used to compare with ALA and ALAENs. All algorithms set the same parameters: the number of iterations is 500, the population size is 30, all algorithms are tested 20 times, and the test results are averaged. When testing CEC2017, 10, 30, 50 and 100-dimensional tests were performed, and ALAEN was the best in all the test results. In this paper, the test results of 30 dimensions are discussed. Standard deviation (std), average fitness value (avg), optimum (min), convergence curve, and ANOVA test will be discussed in detail.

Table 1 and Table 2 show the experimental data results for *F*_1_, *F*_3_ to *F*_10_ and *F*_11_ to *F*_20_ respectively. For the *F*_1_ and *F*_3_ (unimodal) test functions, ALAEN achieved a qualitative leap in parameters such as std, min, and avg compared to ALA and was also much better than the other seven comparison functions. This shows that SDM combined with BetaCOBL enhances the optimization and solution stability of the ALA in the unimodal function test. *F*_4_ to *F*_10_ are simple multi-modal functions. For *F*_4_ to *F*_10_, ALAEN has improved in all parameters compared with ALA and also shows superiority compared with the other 7 algorithms. Compared to other algorithms, ALAEN demonstrates distinct advantages in both average and best fitness values; only its standard deviation is slightly inferior to that of the LSHADE algorithm on a subset of functions. The simple multi-modal function test shows that the joint strategy can enhance the overall performance of the algorithm and make the algorithm have stronger optimization ability and stability. *F*_11_ to *F*_20_ are mixed functions. From the perspective of average fitness value, ALAEN ranks first in the test results on *F*_11_ to *F*_13_, *F*_15_ to *F*_17_, *F*_19_ and *F*_20_ functions. For functions *F*_14_ and *F*_18_ ALAEN ranks second and third in avg parameters. ALAEN also shows excellent performance in terms of standard deviation. The results show that for mixed functions, the joint strategy of SDM and BetaCOBL can improve the optimization ability of the ALA and avoid falling into the local optimal solution too early.

Table 3 shows the test results of functions *F*_21_ to *F*_30_. Functions *F*_21_ to *F*_30_ are composition functions. For functions *F*_21_ to *F*_30_, the avg and min values of ALAEN are significantly improved compared with ALA. The experimental results demonstrate that the proposed ALAEN outperforms the original ALA and 7 state-of-the-art comparison algorithms (EALA, LSHADE, PSO, SSA, GWO, HHO, SFOA) in all three core indicators: min, avg, and std. Especially in high-dimensional complex functions such as F30, the average optimization accuracy of ALAEN is nearly 2 times higher than that of the second-best algorithm, 28 times higher than that of the original ALA, and the stability is improved by nearly 100 times. These results fully verify the effectiveness and universality of the improved strategy of ALAEN, providing a more efficient and robust solution for complex engineering optimization problems. By comparison, it can be seen that in terms of composition functions testing, the ALAEN improved by the joint strategy can greatly enhance the optimization ability and stability of the ALA.

**Figure 4:**
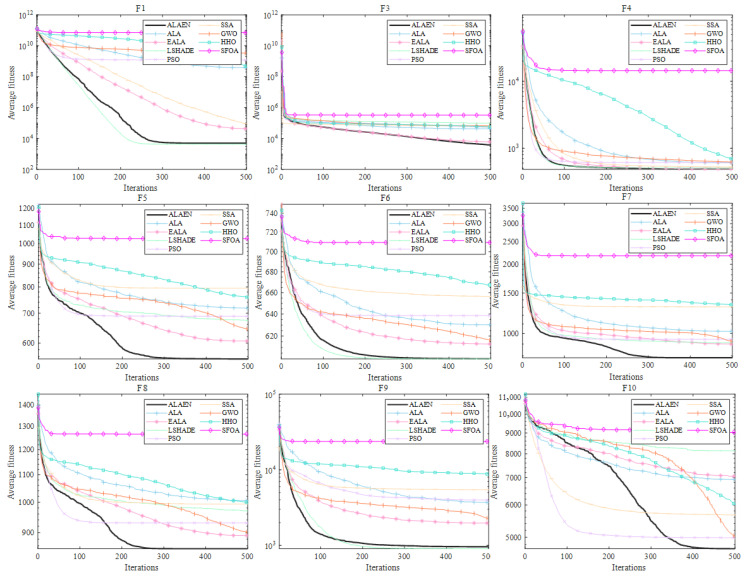
Convergence plot of *F*_1_ and *F*_3_–*F*_10_.

Figure 4 shows the convergence curves of functions *F*_1_ and *F*_3_ to *F*_10_. As shown in Figure 4, the ALA has problems of premature convergence and low iteration efficiency. Its optimization ability ranks in the middle among all algorithms. The experimental results show that the proposed ALAEN achieves the optimal convergence performance in all test functions: on the one hand, ALAEN has the fastest convergence speed, which can decrease rapidly with fewer iterations and avoid premature convergence; on the other hand, ALAEN obtains the lowest final convergence accuracy, outperforming the original ALA and all comparison algorithms comprehensively in unimodal, multimodal, high-dimensional and other types of functions. These results fully verify the effectiveness of the improved strategy of ALAEN, and its global exploration ability, local exploitation ability and robustness are essentially enhanced.

**Figure 5:**
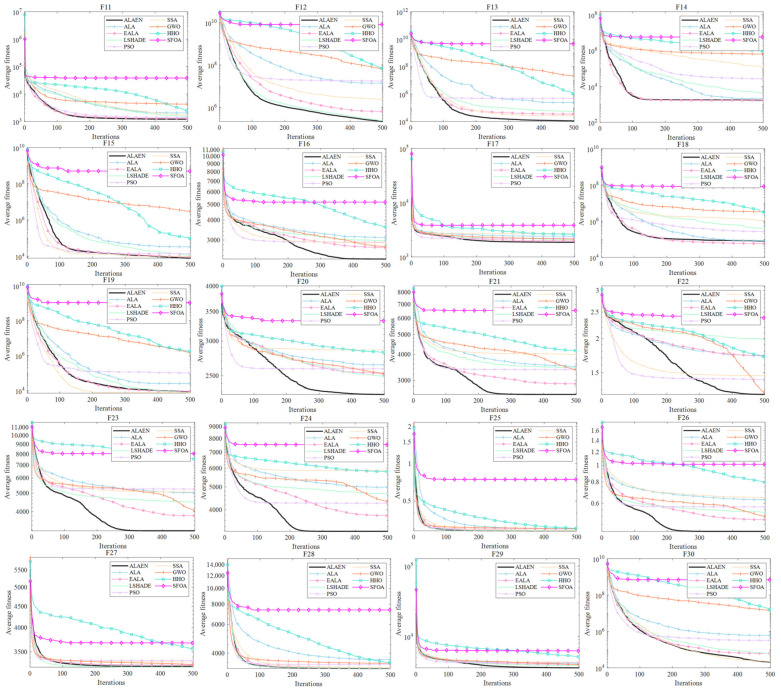
Convergence plot of *F*_11_–*F*_30_.

Figure 5 presents the convergence curves of *F*_11_–*F*_30_. As can be seen from the figure, for functions *F*_11_–*F*_20_, ALAEN has improved in terms of convergence speed and average fitness value compared to ALA. Compared with other algorithms, ALAEN has shown the best performance on most test functions and also performed well on several other test functions. For functions *F*_21_–*F*_30_, from the perspective of average fitness value, ALAEN has shown a clear advantage and ranked first. From the perspective of iteration efficiency, it also ranks high. Compared with the ALA, both the convergence speed and fitness value have been greatly improved. In summary, the ALAEN improved by the joint strategy of BetaCOBL and SDM can greatly improve the optimization ability and iteration efficiency of the ALA on mixed and composition functions.

**Figure 6:**
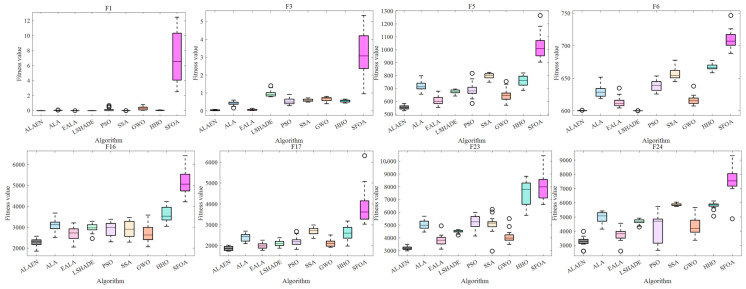
ANOVA test of partial functions in *F*_1_–*F*_30_.

Figure 6 shows the ANOVA test plots of the eight test functions (only representative test functions are selected). The experimental results show that the proposed ALAEN achieves the optimal optimization performance and robustness in all test functions: on the one hand, the box position and median of ALAEN are the lowest among all algorithms, and the optimization accuracy comprehensively outperforms the original ALA and all comparison algorithms; on the other hand, the box height and whisker length of ALAEN are the smallest, with no outliers and extremely concentrated data, demonstrating overwhelming superiority in stability and robustness. These results fully verify the effectiveness of the improved strategy of ALAEN, providing a more efficient and stable solution for complex engineering optimization problems.

Table 4 shows the Wilcoxon results of functions *F*_1_ and *F*_3_ to *F*_30_. In the table, data values less than 0.01 indicate extremely significant differences (strong advantage), data values between 0.01 and 0.05 indicate significant differences (significant advantage), and results greater than 0.05 indicate no significant difference. The test results show that ALAEN’s *p*-values are significantly less than 0.05 on the vast majority of test functions, indicating that the performance difference between ALAEN and the comparative algorithms is statistically significant. Specifically, ALAEN significantly outperforms HHO and SFOA on all 30 functions, significantly outperforms ALA on 29 functions, and is comparable to EALA, LSHADE, PSO, SSA, and GWO on only 2–4 functions. No function shows ALAEN significantly weaker than the comparative algorithms. In conclusion, the optimization performance of the ALAEN has been fully validated statistically, demonstrating significant advantages over existing algorithms and representing a more efficient and robust optimization algorithm.

**Figure 7:**
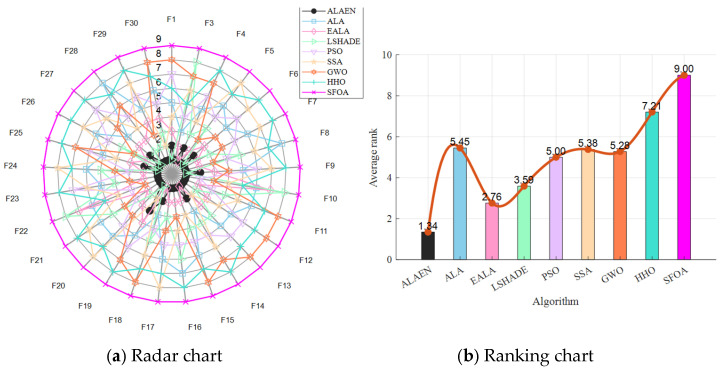
Radar and ranking charts for CEC2017.

Figure 7 shows the radar chart and ranking chart of all algorithms on the CEC2017 test set. As the radar chart shows, the performance curve of the ALAEN almost entirely stays within the central region, achieving best or near-best rankings on the vast majority of test functions. In contrast, the curves of other compared algorithms expand significantly outwards, indicating a substantial performance gap. The average ranking chart shows that ALAEN’s average ranking is only 1.34, a clear lead over all compared algorithms. EALA ranks second with an average ranking of 2.76, followed by LSHADE, PSO, GWO, SSA, and ALA in the middle. HHO and SFOA rank at the bottom, with SFOA having the worst overall performance at an average ranking of 9.00. In conclusion, the ALAEN demonstrates exceptional stability and superiority across all test functions, comprehensively outperforming all compared algorithms and making it the best optimization algorithm in this evaluation.

### 3.2. UAV Track Test Results

#### 3.2.1. Small Map Simulation Scene

In the first scenario, the map size is 230 km × 230 km × 10 km, the starting coordinates of the drone are (20, 20, 20), and the end coordinates are (180, 180, 20). There are two threat areas with coordinates of (150, 50) and (50, 150), and the radius of the threat areas is 30 km and 20 km respectively. The maximum turning angle is 90°, and the values of *w*_1_, *w*_2_ and *w*_3_ are 0.4, 0.4 and 0.2 respectively. The population size was set to 20, the maximum number of iterations was set to 100, and the experiment was repeated 20 times.

**Figure 8:**
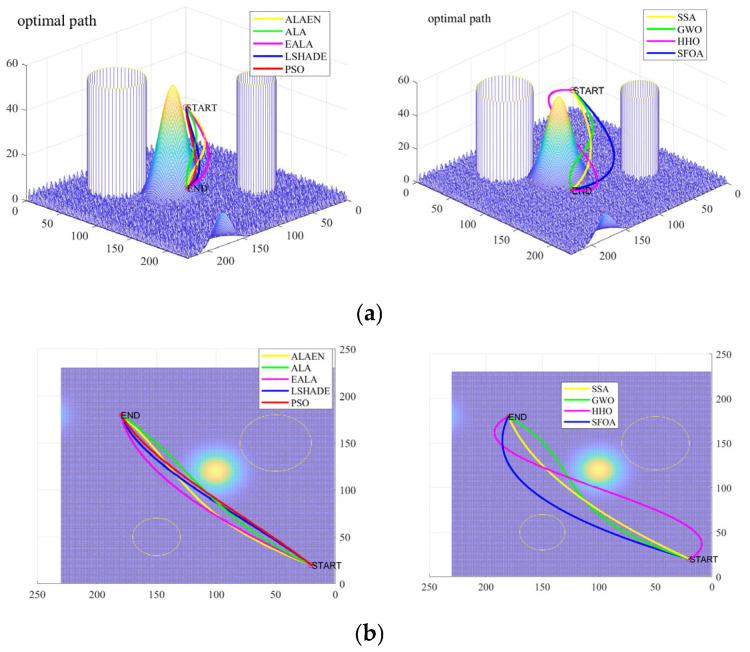
3D and 2D track planning map (small map): (**a**) 3D and (**b**) 2D.

Figure 8 shows a comparison of three-dimensional and two-dimensional drone trajectory planning in a small map context. As shown in Figure 8, the ALAEN produces the path that is closest to the ground, shortest in length, and smoothest, achieving near-straight-line planning from start to finish while perfectly avoiding all obstacles. The LSHADE and ALAs perform slightly worse, while the EALA, PSO, GWO, and SSAs exhibit significant issues such as detours, excessive height, and poor smoothness. The HHOs and SFOAs have the longest paths and the most severe detours, completely failing to meet practical requirements. In conclusion, the ALAEN demonstrates superior performance in complex 3D path planning tasks and is the best path planning algorithm among all compared algorithms.

**Figure 9:**
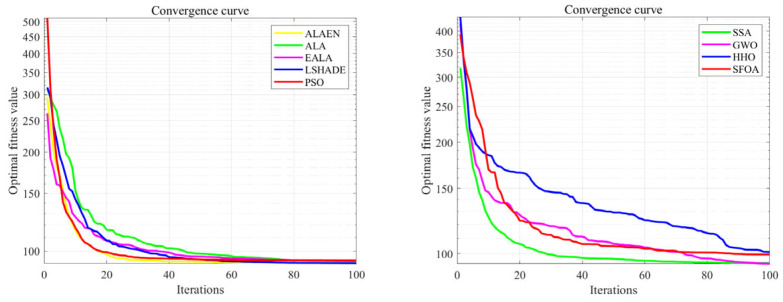
Track evaluation value changes with iterations (small map).

Figure 9 shows the change in the track evaluation function as the number of iterations increases. Compared with ALA, ALAEN has an improvement in optimization ability. Its iteration speed is faster and the optimal cost function value is smaller. It reaches about 91 in about 60 iterations, while the average value of ALA is about 93. Compared with other algorithms, it can be seen from the convergence curve that ALAEN has the smallest average cost function value, and its iteration efficiency is only lower than that of the PSO algorithm.

Table 5 shows the relevant parameters of UAV trajectory planning. As shown in Table 5, the ALAEN has the best average path length (230.885), average flight altitude (91.598), and standard deviation (0.558) globally. Only the shortest path length (90.596) is slightly higher than PSO. Its overall performance is significantly superior to all the compared algorithms. The performance of algorithms such as LSHADE, ALA, and EALA is second best. Traditional algorithms such as PSO, GWO, SSA, HHO, and SFOA have obvious defects in path length, altitude control, and stability. Among them, HHO and SFOA have the worst overall performance. It can be seen that the ALAEN improved by combining SDM and BetaCOBL can greatly improve the optimization ability and stability of the ALA and can be used for UAV trajectory planning in small map scenarios.

#### 3.2.2. Large Map Simulation Scene

In the second scenario, the map size is 450 km × 450 km × 10 km, the starting coordinates of the drone are (20, 20, 20), and the end coordinates are (420, 320, 20). The threat area coordinates and threat radius are shown in Table 6. Other parameter settings remain consistent with the small map simulation scene.

**Figure 10:**
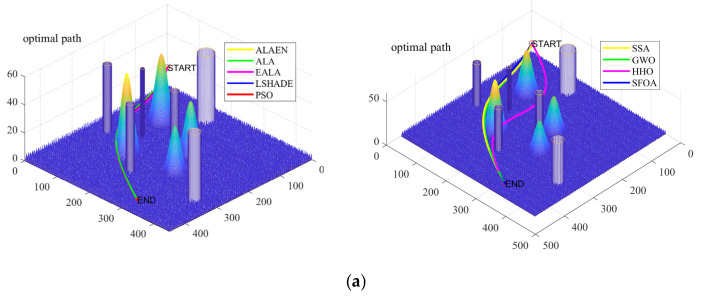

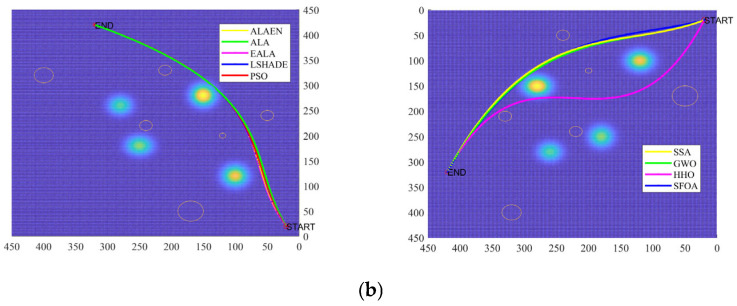
3D and 2D track planning map: (**a**) 3D and (**b**) 2D.

**Figure 11:**
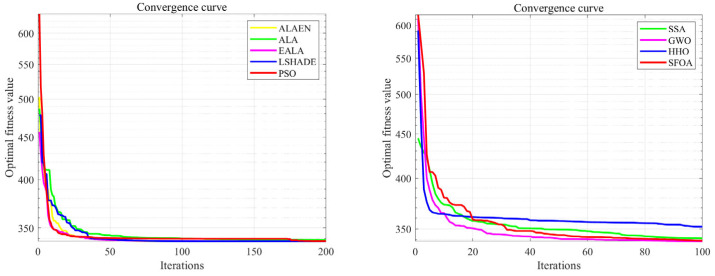
Track evaluation value changes with iterations.

Figure 10 shows a comparison of three-dimensional and two-dimensional drone trajectory planning in a big map context. As can be seen from the figure, the trajectory routes found by all algorithms avoid the threat area. The optimal trajectory routes found by ALAEN, LSHADE and PSO are similar, with shorter lengths and smaller turning angles. The path lengths found by ALA and SSA are greater than ALAEN, and the turning angles are larger. The path found by HHO is the longest and has the largest turning angles. Figure 11 shows the change in the cost function value of the trajectory planning in the large map scenario as the number of iterations increases. The ALAEN has the smallest cost function value, which reaches about 337 in the 60th iteration, and its early iteration efficiency is also significantly higher than that of the ALA. Compared with other functions, its cost function value is the smallest and has a faster iteration efficiency.

Table 7 shows the relevant parameters of UAV trajectory planning in a large map. Table 7 shows that the ALAEN has a globally optimal average path length (527.6914), average flight altitude (337.369), and standard deviation (0.00003). Its shortest path length is tied for first place with top-performing algorithms, and its overall performance surpasses all compared algorithms. Algorithms such as LSHADE and EALA perform second best, while traditional algorithms like PSO, GWO, SSA, HHO, and SFOA all exhibit significant deficiencies in path length, altitude control, and stability. Among these, HHO’s overall performance is the worst. In summary, in large map scenarios, the ALAEN has strong stability, fast convergence speed, and strong optimization ability and shows superiority compared to other algorithms. Therefore, it can be shown that the joint strategy can greatly improve the algorithm’s optimization ability and stability, avoiding premature convergence and falling into the local optimal solution.

## 4. Conclusions and Future Work

This paper studies the problem of three-dimensional UAV trajectory planning, considering the influence of factors such as the shortest path, flight altitude and corner cost on the optimal path of the UAV, and constructs a trajectory evaluation function. This paper first improves the artificial lemming optimization algorithm by introducing stochastic differential mutation and Beta opposition-based learning and conducts detailed test analysis on the CEC2017 test set. The results show that the ALAEN obtained by joint strategy improvement greatly improves the global optimization ability and convergence speed of the original algorithm and effectively avoids falling into the local optimal solution. Compared with other algorithms, the ALA ranks 5.45 and the ALAEN ranks 1.34, which has been greatly improved. Subsequently, the improved ALAEN is used for three-dimensional UAV trajectory planning, and two complex terrains are constructed for path optimization. The comparison of multiple algorithms shows that ALAEN can find the shortest path and has the smallest evaluation function value. The standard deviation values of ALAEN in the two maps are 0.558 and 0.00003, respectively, which are the smallest among all algorithms, indicating its superior stability. In summary, the ALAEN improved by the joint strategy has excellent optimization ability and stability and can be used for three-dimensional UAV trajectory planning problems.

Although the ALAEN proposed in this study demonstrates encouraging performance, several limitations and open research directions remain to be further explored. First, the current evaluation—conducted solely within static test environments containing fixed obstacles—cannot yet fully capture the complexities of real-world UAV path planning. Consequently, future work will focus on evaluating and optimizing the ALAEN framework within dynamic environments that feature moving obstacles (e.g., dynamic traffic flows or moving ground targets), thereby validating its real-time obstacle avoidance capabilities and robustness under time-varying constraints. Second, to bridge the gap between simulation and practical application, subsequent efforts will be dedicated to integrating this path planner with real-time UAV control systems. Comprehensive field experiments will then be conducted on physical platforms to verify its feasibility, computational efficiency, and control performance during actual flight missions. Third, given that the current study focuses on single-UAV path planning—while scenarios such as search and rescue, reconnaissance, and logistics delivery increasingly demand multi-UAV collaborative operations—future research will extend ALAEN to the domain of collaborative multi-UAV path planning, with a specific emphasis on inter-UAV collision avoidance mechanisms, task allocation strategies, and global path optimization problems. Furthermore, future work will aim to enhance ALAEN’s adaptability to complex environmental constraints (e.g., dynamic weather conditions and no-fly zones), thereby expanding its scope of application in more challenging real-world missions.

## Figures and Tables

**Table 1:** Statistical results for *F*_1_ and *F*_3_ to *F*_10_.

*f*	Results	ALAEN	ALA	EALA	LSHADE	PSO	SSA	GWO	HHO	SFOA
*F*_1_	min	144.437	152,329,397.6	5187.25	207.14	7,270,163.17	23,694.76	516,434,249.1	100,841,984	25,245,103,362
	std	4211.81	179,067,588.9	40,551.45	4247.57	2,071,931,045	59,856.73	2,494,608,770	250,173,631.1	33,157,812,099
	avg	4949.71	386,647,253.5	42,347.69	4188.07	1,190,875,144	85,628.13	3,295,219,625	442,321,885	69,667,443,434
	avgti	0.0121	0.0371	0.0113	0.0730	0.0304	0.0504	0.0547	0.0576	0.0524
*F*_3_	min	801.88	15,840.35	1589.03	78,608.37	28,712.11	48,991.36	39,845.27	43,760.38	95,524.78
	std	2173.00	11,800.81	3785.68	16,620.90	18,526.68	7331.35	12,437.11	6570.93	122,105.91
	avg	3713.54	42,138.97	5971.01	95,096.98	53,214.54	59,785.60	65,452.92	54,937.50	322,041.27
	avgti	0.0126	0.0409	0.0117	0.0901	0.0348	0.0558	0.0585	0.0720	0.0558
*F*_4_	min	469.70	539.12	408.54	473.83	524.76	479.22	507.01	515.87	4805.49
	std	18.79	53.22	29.94	14.62	121.46	21.21	83.48	84.16	8185.61
	avg	494.22	607.37	490.99	502.25	612.41	523.84	624.31	694.30	14,465.52
	avgti	0.0127	0.0380	0.0113	0.0733	0.0297	0.0510	0.0548	0.1699	0.1449
*F*_5_	min	526.86	654.51	552.05	640.73	583.18	747.75	569.54	684.59	903.61
	std	14.97	36.89	34.21	14.32	54.30	23.34	45.18	39.95	97.78
	avg	553.14	718.19	606.23	674.00	688.77	795.36	645.35	758.71	1025.76
	avgti	0.0123	0.0401	0.0323	0.2511	0.0929	0.2038	0.1452	0.2229	0.1370
*F*_6_	min	600.01	618.58	604.22	600.00	625.65	644.95	607.24	658.45	688.29
	std	0.20	9.04	7.67	0.01	8.61	9.18	7.12	4.46	13.16
	avg	600.15	630.19	613.12	600.01	638.52	656.52	616.11	667.17	709.38
	avgti	0.0353	0.1441	0.0367	0.2395	0.1245	0.2509	0.1805	0.3105	0.1848
*F*_7_	min	758.59	957.35	836.25	874.80	860.78	1211.02	870.93	1183.63	1427.91
	std	14.75	44.25	41.01	14.47	57.00	40.78	48.11	68.96	760.68
	avg	784.16	1021.28	899.96	909.56	942.55	1306.03	924.52	1333.46	2177.16
	avgti	0.0344	0.1217	0.0126	0.1213	0.0343	0.0581	0.0609	0.0770	0.0586
*F*_8_	min	827.86	937.89	840.84	927.00	887.60	941.29	875.84	955.12	1163.56
	std	18.38	37.39	27.41	18.83	29.21	22.59	20.01	34.83	70.50
	avg	851.09	1005.69	890.73	970.08	930.53	993.15	901.58	998.69	1265.96
	avgti	0.0128	0.0455	0.0113	0.0950	0.0331	0.0549	0.0583	0.0704	0.0786
*F*_9_	min	904.52	1975.36	1109.58	900.00	2259.83	5261.13	1509.35	5580.47	11,378.83
	std	102.45	1443.29	982.05	8.58	1179.81	97.67	605.18	1324.94	7492.21
	avg	959.48	3704.67	1967.16	903.31	3978.00	5435.73	2270.84	8786.13	23,668.65
	avgti	0.0395	0.1431	0.0349	0.2591	0.1169	0.2329	0.1703	0.2808	0.1748
*F*_10_	min	3430.17	5672.28	4791.04	7504.76	3907.99	4540.73	3806.03	4913.45	7976.96
	std	467.99	803.27	1207.65	360.52	520.87	684.08	1014.79	693.59	399.26
	avg	4687.85	6932.34	7053.13	8133.99	4987.23	5669.86	5028.70	6014.15	9033.72
	avgti	0.0388	0.1338	0.0369	0.2681	0.1075	0.2364	0.1639	0.2608	0.1635

**Table 2:** Statistical results for *F*_11_ to *F*_20_.

*f*	Results	ALAEN	ALA	EALA	LSHADE	PSO	SSA	GWO	HHO	SFOA
*F*_11_	min	1148.23	1667.62	1228.48	1370.61	1256.56	1444.29	1583.40	1740.16	12,192.01
	std	60.90	446.64	56.29	111.68	161.84	222.16	1471.17	756.83	15,446.09
	avg	1209.63	2064.75	1295.07	1542.07	1401.97	1695.19	4290.05	2485.85	38,332.50
	avgti	0.0337	0.1136	0.0310	0.2978	0.0816	0.2068	0.1458	0.2112	0.1488
*F*_12_	min	35,025.00	2,571,397.87	74,906.34	32,604.96	459,323.08	152,873.18	6,277,197.88	4,263,009.23	1,442,526,892
	std	224,075.88	8,957,279.49	557,486.59	192,832.54	47,810,980.58	1,789,874.59	61,976,456.92	63,137,717.36	4,047,209,943
	avg	215,029.06	13,855,079.80	643,832.75	233,965.04	17,301,464.14	2,343,019.97	67,231,768.11	66,929,388.28	8,158,483,271
	avgti	0.0356	0.1217	0.0338	0.2369	0.0952	0.2129	0.1545	0.2382	0.1548
*F*_13_	min	1491.80	11,919.23	6239.62	9904.80	9053.07	6819.77	54,331.05	322,395.85	561,220,507
	std	10,485.66	592,264.45	24,128.11	29,987.67	1,369,589.05	24,317.30	66,097,871.55	702,174.34	4,237,522,243
	avg	11,057.57	238,852.85	34,790.62	52,350.18	482,038.49	26,943.54	21,105,721.75	1,056,851.77	4,604,482,114
	avgti	0.0322	0.1195	0.0336	0.2881	0.0981	0.2212	0.1477	0.2341	0.1564
*F*_14_	min	1548.88	1684.61	1555.42	1793.19	2357.51	5295.61	18,795.97	55,338.30	642,341.67
	std	230.12	399.00	65.81	3849.19	24,929.71	91,782.75	625,760.25	868,391.64	4,490,600.04
	avg	1677.71	2061.43	1674.31	4272.90	26,771.86	124,178.23	640,554.43	910,201.39	5,731,234.90
	avgti	0.0350	0.1243	0.0366	0.3137	0.0981	0.2199	0.1576	0.2499	0.1569
*F*_15_	min	2076.14	6341.13	2440.78	3623.40	2739.59	1806.38	20,635.89	18,065.71	18,459,787.56
	std	7241.56	28,668.61	8482.85	7115.33	11,746.41	9268.69	8,260,617.93	47,860.82	534,271,152
	avg	7811.31	32,971.71	9509.48	11,426.97	13,713.24	9954.42	3,003,832.07	94,870.37	495,067,498
	avgti	0.0342	0.0995	0.0328	0.2893	0.0919	0.2095	0.1446	0.2319	0.1485
*F*_16_	min	1862.20	2509.74	2056.56	2462.04	2306.08	2292.40	2069.39	3046.27	4216.34
	std	179.18	314.84	315.63	212.32	339.79	358.12	436.98	374.95	619.79
	avg	2283.39	3116.80	2688.29	2977.16	2910.27	2892.62	2725.33	3616.97	5177.83
	avgti	0.0361	0.0449	0.0340	0.1299	0.0567	0.2228	0.1547	0.2506	0.1556
*F*_17_	min	1739.67	2098.13	1776.66	1879.92	1824.78	2349.44	1924.39	1980.67	3033.12
	std	102.95	193.81	131.99	127.17	212.00	185.85	164.84	334.56	790.72
	avg	1861.25	2398.61	1986.46	2105.12	2198.79	2697.18	2128.08	2612.37	3830.59
	avgti	0.0396	0.1524	0.0384	0.1541	0.1173	0.1485	0.1353	0.3087	0.1833
*F*_18_	min	20,059.75	30,681.40	16,214.40	104,217.75	54,234.28	126,858.73	294,921.51	59,544.70	1,671,118.82
	std	77,718.49	42,476.78	49,745.02	231,026.73	231,271.80	1,048,545.31	5,947,055.08	3,631,936.16	73,046,102
	avg	80,015.99	83,914.22	58,103.09	396,433.46	258,141.84	1,065,516.47	3,274,154.44	3,042,378.77	82,498,349
	avgti	0.0354	0.1193	0.0328	0.2461	0.0853	0.2197	0.1507	0.2291	0.1546
*F*_19_	min	2209.37	2506.85	2237.99	2224.92	2161.63	2206.49	27,703.38	48,084.99	12,553,260
	std	8118.27	19,576.49	5590.05	8075.47	254,923.29	7297.47	1,933,455.64	1,790,857.29	1,431,595,071
	avg	8575.00	24,615.03	8828.28	7094.49	100,493.20	7585.33	1,450,173.33	1,677,844.70	1,061,923,313
	avgti	0.0439	0.1139	0.0521	0.4128	0.2242	0.3845	0.2776	0.5498	0.2849
*F*_20_	min	2034.66	2344.47	2256.03	2289.33	2242.90	2549.94	2268.92	2451.77	2996.69
	std	131.99	157.68	167.08	135.83	195.80	234.16	181.67	219.86	216.22
	avg	2269.60	2647.39	2526.45	2484.49	2593.17	2862.50	2532.21	2827.65	3335.46
	avgti	0.0431	0.1592	0.0385	0.3117	0.1316	0.2633	0.1843	0.3258	0.1933

**Table 3:** Statistical results for *F*_21_ to *F*_30_.

*f*	Results	ALAEN	ALA	EALA	LSHADE	PSO	SSA	GWO	HHO	SFOA
*F*_21_	min	2200.00	2268.29	2200.90	2200.00	2260.72	2202.89	2267.26	2322.55	3675.75
	std	341.06	1025.96	612.89	825.87	779.73	936.96	460.28	670.05	1091.73
	avg	2565.79	3503.86	2882.49	3446.26	3378.73	4008.15	3380.32	4166.34	6531.92
	avgti	0.0418	0.1711	0.0404	0.3093	0.1451	0.2740	0.1972	0.3486	0.2074
*F*_22_	min	9682.98	11,391.90	10,802.13	18,510.26	10,449.64	9736.86	9536.61	14,276.74	22,300.77
	std	1932.99	2600.28	3081.93	823.91	2210.95	1749.39	3090.76	2194.52	651.74
	avg	12,519.03	17,295.39	17,321.81	19,891.07	14,164.70	14,639.35	12,717.11	17,106.66	23,811.57
	avgti	0.0457	0.1944	0.0471	0.3219	0.1641	0.3052	0.2273	0.4183	0.2315
*F*_23_	min	2978.61	4451.63	3114.28	4219.59	4141.98	2959.51	3485.38	5758.43	6610.44
	std	134.67	389.56	410.52	116.79	465.58	638.28	453.34	934.02	1023.89
	avg	3182.49	5027.86	3816.18	4495.10	5255.84	5060.77	4078.02	7540.54	8053.74
	avgti	0.0479	0.2138	0.0479	0.3453	0.1775	0.3257	0.2422	0.4388	0.2446
*F*_24_	min	2600.00	4147.52	2601.92	4295.23	2650.76	5743.02	3363.60	5057.80	4873.63
	std	342.90	376.94	424.66	173.79	980.27	80.10	652.33	223.63	870.62
	avg	3248.99	4986.36	3782.75	4674.67	4268.18	5867.96	4341.80	5803.84	7545.08
	avgti	0.0463	0.2030	0.0451	0.3323	0.1728	0.3087	0.2286	0.4239	0.2359
*F*_25_	min	2878.64	2948.04	2880.25	2878.64	2891.01	2878.96	2926.84	2971.08	3739.29
	std	6.23	28.73	8.15	3.32	57.21	15.64	52.08	31.39	2970.83
	avg	2881.88	2984.49	2886.00	2881.64	2991.18	2898.18	2999.76	3012.36	7478.13
	avgti	0.0465	0.2035	0.0456	0.3505	0.1778	0.3238	0.2323	0.4032	0.2374
*F*_26_	min	2800.00	5141.40	2807.12	5013.20	3451.01	2805.11	4299.88	3481.83	8722.72
	std	355.91	562.17	585.63	218.65	1291.39	2058.69	490.95	1403.02	1103.08
	avg	4106.16	6293.78	4808.95	5296.45	5711.59	6473.99	5036.81	7931.72	10,154.49
	avgti	0.0527	0.2334	0.0502	0.3028	0.1960	0.3395	0.2599	0.4880	0.2688
*F*_27_	min	3205.18	3212.28	3201.77	3201.38	3261.45	3225.40	3224.08	3322.20	3407.24
	std	17.47	32.34	30.74	9.75	52.42	75.43	25.01	180.29	153.64
	avg	3229.77	3253.78	3250.55	3215.59	3335.97	3310.55	3264.94	3559.16	3674.68
	avgti	0.0528	0.2396	0.0506	0.3500	0.2149	0.3004	0.2315	0.4382	0.2818
*F*_28_	min	3199.91	3312.35	3200.45	3199.45	3253.12	3209.24	3360.01	3344.55	5693.33
	std	16.92	741.17	20.29	21.00	117.98	21.52	88.34	116.45	1160.39
	avg	3219.29	3647.81	3234.36	3229.23	3406.91	3259.48	3460.86	3490.53	7374.87
	avgti	0.0498	0.2228	0.0479	0.3672	0.1909	0.3395	0.2514	0.4490	0.2577
*F*_29_	min	3391.13	3664.78	3504.53	3547.81	3781.39	3692.14	3584.44	4243.92	5274.32
	std	208.11	235.43	316.13	167.10	308.82	324.44	276.68	536.12	670.49
	avg	3581.95	4173.60	4028.45	3858.50	4256.89	4310.76	3985.73	5133.93	6251.80
	avgti	0.0474	0.2064	0.0457	0.3489	0.1811	0.3248	0.2348	0.4347	0.2486
*F*_30_	min	5598.91	21,287.72	10,790.58	16,373.14	22,500.41	9801.31	2,180,048.23	2,413,733.80	138,294,194.57
	std	12,961.04	1,170,871.46	106,266.66	43,363.08	564,061.03	8536.67	8,748,600.92	8,498,285.39	493,732,710.21
	avg	21,169.13	612,013.19	61,806.74	59,281.82	321,372.73	22,282.77	14,169,336.57	13,488,329.11	691,048,727.94
	avgti	0.0610	0.2915	0.0592	0.4501	0.2723	0.4422	0.3266	0.6504	0.3384

**Table 4:** Wilcoxon results for *F*_1_ and *F*_3_ to *F*_30_.

	ALA	EALA	LSHADE	PSO	SSA	GWO	HHO	SFOA
*F*_1_	6.796 × 10^−8^	1.803 × 10^−6^	5.609 × 10^−1^	6.796 × 10^−8^	6.796 × 10^−8^	6.796 × 10^−8^	6.796 × 10^−8^	6.796 × 10^−8^
*F*_3_	6.796 × 10^−8^	4.986 × 10^−2^	6.796 × 10^−8^	6.796 × 10^−8^	6.796 × 10^−8^	6.796 × 10^−8^	6.796 × 10^−8^	6.796 × 10^−8^
*F*_4_	6.796 × 10^−8^	9.246 × 10^−1^	2.733 × 10^−1^	6.796 × 10^−8^	2.041 × 10^−5^	2.960 × 10^−7^	1.065 × 10^−7^	6.796 × 10^−8^
*F*_5_	6.796 × 10^−8^	1.047 × 10^−6^	6.796 × 10^−8^	6.796 × 10^−8^	6.796 × 10^−8^	1.065 × 10^−7^	6.796 × 10^−8^	6.796 × 10^−8^
*F*_6_	6.796 × 10^−8^	6.796 × 10^−8^	4.539 × 10^−7^	6.796 × 10^−8^	6.796 × 10^−8^	6.796 × 10^−8^	6.796 × 10^−8^	6.796 × 10^−8^
*F*_7_	6.796 × 10^−8^	6.796 × 10^−8^	6.796 × 10^−8^	6.796 × 10^−8^	6.796 × 10^−8^	6.796 × 10^−8^	6.796 × 10^−8^	6.796 × 10^−8^
*F*_8_	6.796 × 10^−8^	9.748 × 10^−6^	6.796 × 10^−8^	1.431 × 10^−7^	6.796 × 10^−8^	6.917 × 10^−7^	6.796 × 10^−8^	6.796 × 10^−8^
*F*_9_	6.796 × 10^−8^	3.416 × 10^−7^	6.015 × 10^−7^	6.796 × 10^−8^	6.796 × 10^−8^	6.796 × 10^−8^	6.796 × 10^−8^	6.796 × 10^−8^
*F*_10_	6.796 × 10^−8^	5.227 × 10^−7^	6.796 × 10^−8^	1.017 × 10^−1^	1.600 × 10^−5^	2.393 × 10^−1^	7.948 × 10^−7^	6.796 × 10^−8^
*F*_11_	6.796 × 10^−8^	3.382 × 10^−4^	6.796 × 10^−8^	3.069 × 10^−6^	6.796 × 10^−8^	6.796 × 10^−8^	6.796 × 10^−8^	6.796 × 10^−8^
*F*_12_	6.796 × 10^−8^	1.481 × 10^−3^	4.570 × 10^−1^	1.235 × 10^−7^	4.539 × 10^−7^	6.796 × 10^−8^	6.796 × 10^−8^	6.796 × 10^−8^
*F*_13_	5.227 × 10^−7^	2.222 × 10^−4^	3.069 × 10^−6^	6.674 × 10^−6^	3.057 × 10^−3^	6.796 × 10^−8^	6.796 × 10^−8^	6.796 × 10^−8^
*F*_14_	3.499 × 10^−6^	6.389 × 10^−2^	3.939 × 10^−7^	7.898 × 10^−8^	6.796 × 10^−8^	6.796 × 10^−8^	6.796 × 10^−8^	6.796 × 10^−8^
*F*_15_	7.577 × 10^−6^	5.075 × 10^−1^	3.372 × 10^−2^	4.986 × 10^−2^	3.942 × 10^−1^	7.898 × 10^−8^	1.431 × 10^−7^	6.796 × 10^−8^
*F*_16_	9.173 × 10^−8^	6.610 × 10^−5^	1.065 × 10^−7^	1.201 × 10^−6^	9.127 × 10^−7^	1.997 × 10^−4^	6.796 × 10^−8^	6.796 × 10^−8^
*F*_17_	6.796 × 10^−8^	1.625 × 10^−3^	2.690 × 10^−6^	1.201 × 10^−6^	6.796 × 10^−8^	1.104 × 10^−5^	1.235 × 10^−7^	6.796 × 10^−8^
*F*_18_	2.733 × 10^−1^	8.103 × 10^−2^	6.917 × 10^−7^	7.406 × 10^−5^	3.416 × 10^−7^	7.898 × 10^−8^	3.939 × 10^−7^	6.796 × 10^−8^
*F*_19_	1.953 × 10^−3^	5.250 × 10^−1^	5.609 × 10^−1^	8.604 × 10^−1^	7.353 × 10^−1^	7.898 × 10^−8^	6.796 × 10^−8^	6.796 × 10^−8^
*F*_20_	1.376 × 10^−6^	4.166 × 10^−5^	7.406 × 10^−5^	1.104 × 10^−5^	6.796 × 10^−8^	5.255 × 10^−5^	9.173 × 10^−8^	6.796 × 10^−8^
*F*_21_	5.115 × 10^−3^	9.786 × 10^−3^	4.320 × 10^−3^	5.629 × 10^−4^	4.166 × 10^−5^	2.356 × 10^−6^	2.062 × 10^−6^	6.796 × 10^−8^
*F*_22_	7.577 × 10^−6^	1.415 × 10^−5^	6.796 × 10^−8^	2.748 × 10^−2^	1.782 × 10^−3^	6.168 × 10^−1^	2.062 × 10^−6^	6.796 × 10^−8^
*F*_23_	6.796 × 10^−8^	2.062 × 10^−6^	6.796 × 10^−8^	6.796 × 10^−8^	1.201 × 10^−6^	7.898 × 10^−8^	6.796 × 10^−8^	6.796 × 10^−8^
*F*_24_	6.796 × 10^−8^	3.705 × 10^−5^	6.796 × 10^−8^	6.557 × 10^−3^	6.796 × 10^−8^	5.227 × 10^−7^	6.796 × 10^−8^	6.796 × 10^−8^
*F*_25_	6.796 × 10^−8^	1.610 × 10^−4^	3.942 × 10^−1^	7.898 × 10^−8^	8.597 × 10^−6^	6.796 × 10^−8^	6.796 × 10^−8^	6.796 × 10^−8^
*F*_26_	6.796 × 10^−8^	3.499 × 10^−6^	6.796 × 10^−8^	3.966 × 10^−3^	8.357 × 10^−4^	2.218 × 10^−7^	1.047 × 10^−6^	6.796 × 10^−8^
*F*_27_	1.143 × 10^−2^	2.390 × 10^−2^	6.040 × 10^−3^	1.065 × 10^−7^	1.576 × 10^−6^	4.680 × 10^−5^	6.796 × 10^−8^	6.796 × 10^−8^
*F*_28_	6.796 × 10^−8^	1.332 × 10^−2^	8.585 × 10^−2^	9.173 × 10^−8^	6.674 × 10^−6^	6.796 × 10^−8^	6.796 × 10^−8^	6.796 × 10^−8^
*F*_29_	6.917 × 10^−7^	2.596 × 10^−5^	1.610 × 10^−4^	7.948 × 10^−7^	4.539 × 10^−7^	2.596 × 10^−5^	6.796 × 10^−8^	6.796 × 10^−8^
*F*_30_	3.416 × 10^−7^	6.557 × 10^−3^	4.680 × 10^−5^	2.356 × 10^−6^	3.648 × 10^−1^	6.796 × 10^−8^	6.796 × 10^−8^	6.796 × 10^−8^

**Table 5:** UAV path planning-related parameters (small map).

	ALAEN	ALA	EALA	LSHADE	PSO	SSA	GWO	HHO	SFOA
apl	230.885	232.559	238.467	234.277	236.956	235.204	231.168	243.141	245.049
min	90.596	90.713	91.568	90.906	90.495	90.71	91.68	93.35	93.00
avg	91.598	92.999	93.222	91.669	93.498	94.24	93.84	101.05	99.45
std	0.558	2.355	1.116	0.845	2.319	3.012	1.583	7.115	3.977

**Table 6:** Threat area distribution.

Sequence	Threat Zone Coordinates	Threat Radius
1	(50, 170)	20
2	(330, 210)	10
3	(240, 50)	10
4	(320, 400)	15
5	(200, 120)	5
6	(220, 240)	10

**Table 7:** UAV path planning-related parameters.

	ALAEN	ALA	EALA	LSHADE	PSO	SSA	GWO	HHO	SFOA
apl	527.6914	528.598	538.5516	527.6938	527.7622	541.5114	530.739	565.6836	544.6144
min	337.369	337.381	337.369	337.369	337.369	339.133	338.143	339.565	338.244
avg	337.369	338.769	337.377	337.369	337.613	341.243	338.564	351.993	339.224
std	0.00003	2.0643	0.0103	0.00014	0.5352	6.357	0.374	6.899	0.724

## Data Availability

The original contributions presented in this study are included in the article. Further inquiries can be directed to the corresponding author.
